# Early systemic immune-inflammation index is associated with mortality and functional outcome in brainstem hemorrhage

**DOI:** 10.3389/fneur.2026.1879981

**Published:** 2026-08-26

**Authors:** Song He, Youkun Zhang, Wenfeng Wei, Huiyang Shi, Rong Qin, Zhenxing Yu, Xianbing Zhang, Lei Deng, Yongjie Zou

**Affiliations:** 1 Department of Neurosurgery, No 908th Hospital of Chinese PLA Joint Logistic Support Force, Nanchang, Jiangxi, China; 2 Department of Neurosurgery, Changcheng Hospital Affiliated to Nanchang University, Nanchang, Jiangxi, China; 3 Department of Radiology, No 908th Hospital of Chinese PLA Joint Logistic Support Force, Nanchang, Jiangxi, China

**Keywords:** brainstem hemorrhage, functional outcome, inflammation, intracerebral hemorrhage, mortality, systemic immune-inflammation index

## Abstract

**Background:**

Brainstem hemorrhage is a severe subtype of spontaneous intracerebral hemorrhage, characterized by high mortality and disability rates, and is closely associated with systemic inflammatory responses. The systemic immune-inflammation index (SII), as a comprehensive inflammatory marker, reflects the balance between immunity and inflammation. However, its dynamic changes and clinical significance in patients with brainstem hemorrhage remain unclear.

**Objective:**

This study aimed to investigate the early dynamic changes in SII in patients with brainstem hemorrhage and to evaluate its association with 30-day mortality and 90-day functional outcomes, thereby providing a potential biomarker for clinical risk assessment.

**Methods:**

We retrospectively enrolled 140 patients with brainstem hemorrhage admitted to the Department of Neurosurgery. SII was calculated from complete blood counts on days 1, 3, and 7 after admission. Patients were divided into high and low SII groups based on the median SII on day 1. Demographic data, medical history, admission GCS score, and blood pressure were collected. Outcomes included 30-day mortality, 90-day modified Rankin Scale (mRS) score, and complications. Linear mixed-effects models were used to analyze SII trajectories, multivariable logistic regression to identify independent predictors, and ROC curve analysis to evaluate predictive performance.

**Results:**

SII was significantly elevated on day 1, peaked on day 3, and declined by day 7. The linear mixed-effects model revealed a significant group-by-time interaction (*p* < 0.001), indicating a more intense and slower-resolving inflammatory response in the high-SII group. The high-SII group had significantly higher rates of 90-day poor functional outcome (*p* < 0.001) and 30-day mortality (*p* = 0.004). Multivariable logistic regression identified Day 3 SII as an independent predictor of both 90-day poor outcome (*p* = 0.011) and 30-day mortality (*p* < 0.001). ROC analysis showed that Day 3 SII predicted 30-day mortality with an AUC of 0.878, and the combined model achieved an AUC of 0.920.

**Conclusion:**

In patients with brainstem hemorrhage, SII is markedly elevated in the early phase, showing a dynamic pattern of rapid rise on day 1, peak on day 3, and gradual decline by day 7. High SII is closely associated with increased mortality and poor functional outcomes, suggesting that SII can serve as an early prognostic biomarker and guide clinical risk stratification and treatment decisions.

## 1 Introduction

Brainstem hemorrhage (BSH) is among the most severe types of spontaneous intracerebral hemorrhage, making up about 5–10% of all cases and linked to high rates of morbidity, mortality, and prolonged hospitalization (1, 2). Due to the brainstem’s compact structure, injuries in this area can quickly disrupt respiratory and circulatory functions, accelerate neurological decline, and significantly increase the risk of death (3). Existing research indicates that early disease progression in BSH patients is closely associated with secondary brain injury, encompassing processes such as inflammatory response, immune dysregulation, blood–brain barrier disruption, and tissue edema. However, there is currently a lack of simple, stable, and reproducible biological markers in clinical practice for the dynamic assessment of the inflammatory state and prognostic risk of BSH (4, 5).

The systemic immune-inflammation index (SII), which integrates platelet, neutrophil, and lymphocyte counts, provides a comprehensive measure of systemic inflammatory activation, immune status, and hypercoagulability (6). Recent studies indicate that SII is closely related to disease severity and outcomes in cerebrovascular diseases and cancers (7). In patients with acute ischemic stroke, higher SII levels correlate with increased stroke severity, early neurological decline, hemorrhagic transformation, and poor functional outcomes. This suggests that SII may be a valuable biomarker for systemic inflammation and vascular injury after an ischemic stroke (8–10). Similarly, in patients with spontaneous intracerebral hemorrhage, elevated SII levels have been associated with poor functional outcomes, neurological deterioration, and increased mortality risk, suggesting its potential value for inflammatory risk stratification after hemorrhage (11, 12).

Elevated SII has been found to correlate with hematoma expansion, early neurological decline, and poor outcomes at 3 months, likely through neutrophil extracellular traps that promote thrombin generation, platelet aggregation, and endothelial damage (11, 13). Beyond cerebrovascular diseases, there is increasing evidence that SII provides valuable prognostic information in various vascular disorders. Higher SII levels are associated with more severe disease in peripheral artery disease, increased complexity of coronary artery lesions, and adverse cardiovascular outcomes in coronary artery disease patients (14–16). Additionally, systemic inflammation-based indices, including SII, have shown potential prognostic significance in patients with abdominal aortic aneurysm, highlighting the role of systemic inflammatory activation in the progression of vascular diseases. These findings suggest that SII may reflect systemic inflammatory activation and vascular immune responses that drive disease progression across different vascular conditions (17).

Compared to individual inflammatory markers, the systemic immune-inflammation index (SII) provides a more comprehensive view of the balance between inflammation and immunity, offering greater sensitivity and prognostic value (18, 19). Unlike traditional inflammatory markers like C-reactive protein and specific leukocyte counts, the SII integrates neutrophil-driven innate immune activation, lymphocyte-related immune suppression, and platelet-linked inflammatory thrombosis. This approach offers a comprehensive evaluation of systemic inflammatory status (20, 21). Additionally, SII’s advantages of low cost, easy accessibility, and reproducibility make it particularly appealing for clinical use in acute neurological disorders (22).

Compared to individual inflammatory markers, the SII provides a more comprehensive view of the balance between inflammation and immunity, offering greater sensitivity and prognostic value (18, 19). Unlike conventional inflammatory indicators such as C-reactive protein and individual leukocyte counts, SII incorporates neutrophil-mediated innate immune activation, lymphocyte-related immune suppression, and platelet-associated inflammatory thrombosis, thereby providing a multidimensional assessment of systemic inflammatory status (20, 21). Additionally, SII’s advantages of low cost, easy accessibility, and reproducibility make it particularly appealing for clinical use in acute neurological disorders (22).

Although SII has demonstrated potential clinical utility in acute ischemic stroke, intracerebral hemorrhage, and stroke-related complications, there is a lack of systematic studies on the early dynamic changes of SII in patients with brainstem hemorrhage and its relationship with secondary brain injury and clinical outcomes (23, 24). Most previous research has concentrated on a single baseline measurement of inflammatory biomarkers, while the inflammatory responses following brainstem hemorrhage are dynamic processes that develop during the acute phase (11, 25). It remains unclear whether serial monitoring of SII offers additional prognostic information beyond conventional clinical parameters, such as the Glasgow Coma Scale score and hematoma volume (26).

Based on this, our study aims to systematically evaluate the early dynamic changes in SII (days 1, 3, and 7 after admission) in patients with brainstem hemorrhage and to explore their association with 30-day mortality and 90-day clinical outcomes. By elucidating the temporal profile and predictive value of SII in brainstem hemorrhage, this study seeks to provide novel insights for early risk stratification and precision intervention in clinical practice.

## 2 Methods

### 2.1 Research patients

This retrospective observational study included 140 patients with brainstem hemorrhage who were admitted to the Department of Neurosurgery at our institution. All patients received appropriate treatment based on the severity of their condition.

Inclusion criteria:

- diagnosis of brainstem hemorrhage confirmed by cranial computed tomography (CT);
- age ≥18 years;
- onset-to-admission time <24 h with available complete blood routine tests;
- complete clinical and follow-up data.

Exclusion criteria:

- history of major stroke, active infection, or autoimmune/immune-related disorders;
- coagulation dysfunction or current use of anticoagulant medication;
- incomplete clinical data.

### 2.2 Clinical and laboratory data collection

Peripheral blood routine parameters were collected on days 1, 3, and 7 after admission to calculate the systemic immune-inflammation index (SII), defined as:


SII=platelet count×neutrophil count/lymphocyte count.

Blood samples from Day 1 were collected within 6 h of admission. Patients were divided into high-SII and low-SII groups based on the median SII value on Day 1 after admission (median = 1,550.70 × 10^9^/L). Those with a Day 1 SII of 1,550.70 × 10^9^/L or higher were classified as high-SII (*n* = 70), while those with a Day 1 SII below this threshold were classified as low-SII (*n* = 70). We also recorded demographic information, medical history, admission Glasgow Coma Scale (GCS) score, hematoma volume, and hematoma location.

The 90-day modified Rankin Scale (mRS) score was used to assess functional outcomes. In addition, complications such as central fever, pulmonary infection, and hyponatremia were recorded. To evaluate the temporal pattern of systemic inflammatory responses, serial SII measurements obtained on Days 1, 3, and 7 were analyzed. In addition, the relationship between SII and hematoma volume was assessed to explore the association between systemic inflammation and hemorrhage severity. To further determine the predictive value of SII for poor outcomes, receiver operating characteristic (ROC) curve analyzes were performed for SII at each time point. The area under the curve (AUC), Youden index, and corresponding optimal cutoff values were calculated. Differences in secondary brain injury severity and clinical outcomes between the high- and low-SII groups were subsequently compared.

### 2.3 Hematoma volume measurement

Hematoma volume was calculated using the widely accepted ABC/2 method, where A represents the greatest hemorrhage diameter on the largest axial CT slice, B represents the diameter perpendicular to A, and C represents the number of CT slices containing hemorrhage multiplied by slice thickness. Hematoma volume was estimated as:


Volume=A×B×C/2.

To reduce measurement bias, hematoma volume was independently assessed by two experienced neuroradiologists who were blinded to the clinical outcomes. Any disagreement was resolved by consensus.

### 2.4 Statistical analysis

Statistical analyzes were performed using SPSS version 27.0 (IBM Corp., Armonk, NY, USA). The normality of continuous variables was assessed using the Kolmogorov–Smirnov test. Variables following a normal distribution were expressed as mean ± standard deviation and compared between groups using the independent-sample *t*-test or one-way analysis of variance. Non-normally distributed variables were presented as median (interquartile range) and compared using the Mann–Whitney U test or Kruskal–Wallis test. Categorical variables were compared using the chi-square test or Fisher’s exact test, as appropriate.

Longitudinal changes in SII were evaluated using a linear mixed-effects model, with group (high-SII vs. low-SII), time (Days 1, 3, and 7), and the group-by-time interaction treated as fixed effects and subject identification included as a random effect. Spearman correlation analysis was performed to evaluate the relationship between serial SII measurements and hematoma volume. Variables with *p* < 0.05 in the univariate analysis or variables considered clinically relevant based on previous literature were subsequently entered into multivariable logistic regression models to identify independent predictors of 90-day poor functional outcome (mRS ≥ 3) and 30-day mortality. ROC curve analysis was performed to evaluate the predictive performance of SII at different time points. The predictive performance of the combined model was further evaluated by calculating the AUC, sensitivity, specificity, and optimal cutoff value based on the Youden index. A two-sided *p* value < 0.05 was considered statistically significant.

## 3 Results

### 3.1 Baseline characteristics

**Figure 1:**
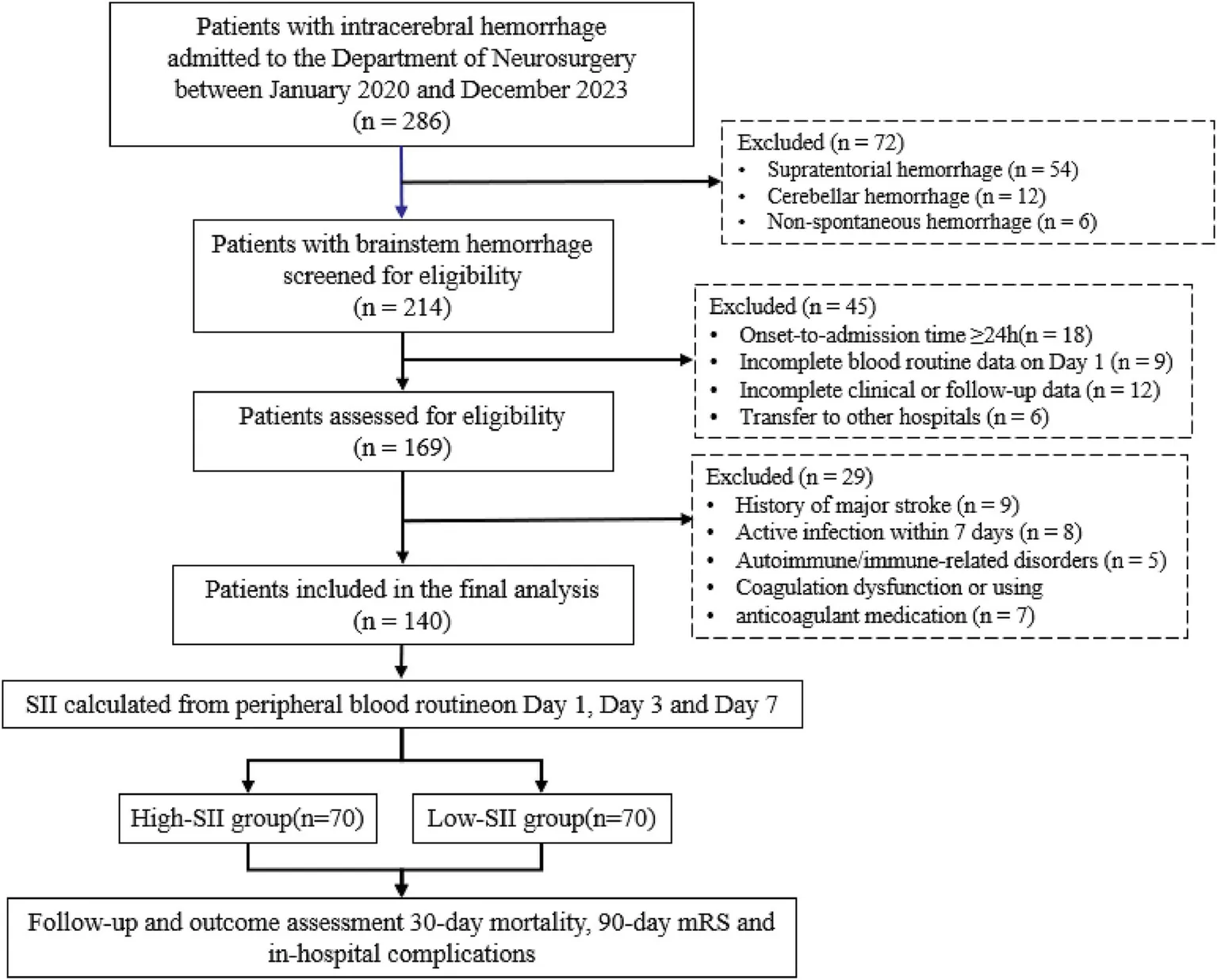
Flowchart of patient enrollment, grouping, and follow-up.

A total of 140 patients with brainstem hemorrhage were enrolled in this study and stratified into a high-SII group (*n* = 70) and a low-SII group (*n* = 70) based on the median SII value measured on Day 1 after admission (Figure 1). There were no significant differences between the two groups in terms of age, sex, history of hypertension or diabetes, smoking or alcohol consumption, systolic/diastolic blood pressure (SBP/DBP), mean arterial pressure (MAP), primary hemorrhage location (pons, midbrain, or medulla), or the incidence of hydrocephalus (all *p* > 0.05). These findings indicate that the baseline clinical characteristics were comparable between the two groups (Table 1).

**Table 1:** Patients’ baseline characteristics.

Characteristic	High SII	Low SII	*p*-value
Age	63.13 ± 9.06	64.09 ± 9.91	0.593
Gender; *n* (%)			
Male	30 (42.9%)	36 (51.4%)	0.310
Female	40 (57.1%)	34 (48.6%)	
SBP	151.5 ± 20.36	145.06 ± 18.14	0.404
DBP	89.4 ± 9.77	88.63 ± 10.76	0.253
MAP	110.1 ± 9.25	107.44 ± 10.25	0.673
GCS	8.63 ± 2.35	10.07 ± 2.71	<0.001
Hypertension	43 (61.4%)	33 (47.1%)	0.09
Diabetes	22 (31.4%)	27 (38.6%)	0.376
Main location of hemorrhage; *n* (%)			
Pons	47 (67.1%)	40 (57.1%)	0.223
Midbrain	12 (17.1%)	16 (22.9%)	0.398
Medulla	11 (15.7%)	14 (20.0%)	0.508
Hydrocephalus	25 (35.7%)	20 (28.6%)	0.366
Smoking	18 (25.7%)	21 (30.0%)	0.572
Alcohol	22 (31.4%)	19 (27.1%)	0.577

### 3.2 SII dynamic changes

**Figure 2:**
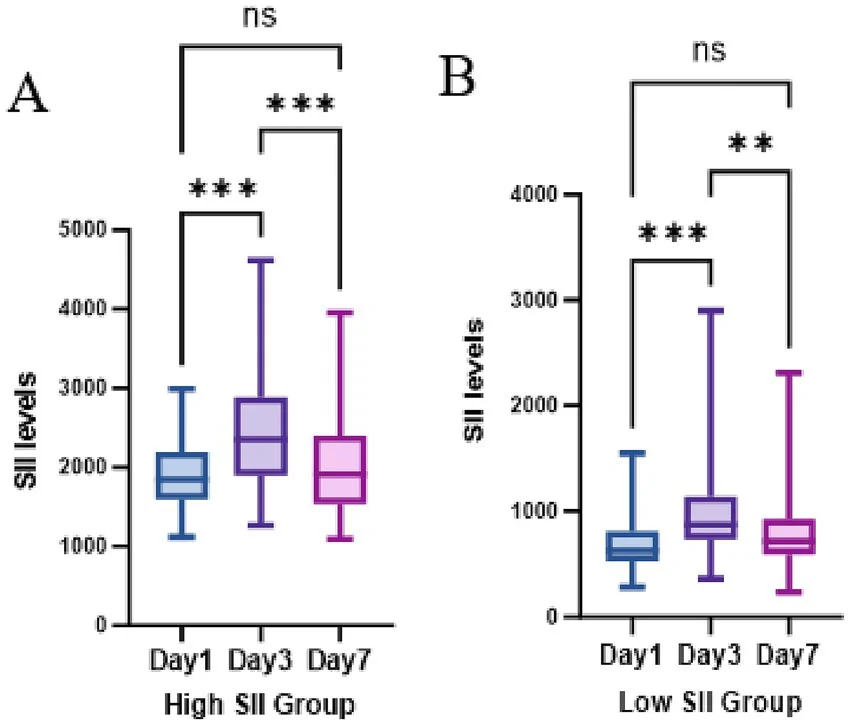
Dynamic changes in SII on Days 1, 3, and 7 after admission in patients with brainstem hemorrhage in high- and low-SII groups. Data are presented as mean ± SD. Statistical comparisons between high- and low-SII groups at each time point were performed using one-way ANOVA. ^*^p < 0.05, ^**^p < 0.01, ^***^p < 0.001. **(A)** High SII group (*n* = 70); **(B)** Low SII group (*n* = 70).

As shown in Table 2, patients in the high-SII group exhibited significantly higher SII values than those in the low-SII group on Day 1 after admission (1,927.98 ± 428.57 vs. 678.51 ± 213.67, *p* < 0.01), indicating that brainstem hemorrhage triggers a pronounced systemic immune-inflammatory response early in the disease course. This response peaked on Day 3, with SII further increasing to 2,473.12 ± 782.00 in the high-SII group, compared with 953.80 ± 377.23 in the low-SII group, representing the most pronounced intergroup difference (*p* < 0.01). This peak may reflect the stage of maximal inflammatory activation or immune dysregulation. By Day 7, although SII values in both groups had declined from their peak levels, the high-SII group (2,072.56 ± 653.31) remained significantly higher than the low-SII group (783.82 ± 315.38, *p* < 0.01), suggesting a slower resolution of systemic inflammation and prolonged immune activation in patients with high SII (Figure 2). Overall, the high-SII group demonstrated a more pronounced early inflammatory response, sustained immune suppression, and heightened platelet activation.

**Table 2:** Dynamic changes of SII at Day 1, Day 3, and Day 7.

Parameter	High SII	Low SII	*p*-value
Day 1			
Neutrophils (×10^9^/L)	8.29 ± 0.91	5.04 ± 1.00	<0.01
Lymphocytes (×10^9^/L)	1.01 ± 0.17	1.53 ± 0.31	<0.05
Platelets (×10^9^/L)	230.29 ± 25.63	197.79 ± 23.84	<0.001
SII (×10^9^/L)	1,915.01 ± 346.02	678.51 ± 213.67	<0.01
Day 3			
Neutrophils (×10^9^/L)	9.29 ± 0.94	6.04 ± 1.21	<0.01
Lymphocytes (×10^9^/L)	0.92 ± 0.21	1.43 ± 0.32	<0.01
Platelets (×10^9^/L)	231.94 ± 34.51	211.71 ± 26.61	<0.05
SII (×10^9^/L)	2,473.12 ± 782.00	953.80 ± 377.23	<0.01
Day 7			
Neutrophils (×10^9^/L)	8.43 ± 0.98	5.28 ± 1.24	<0.05
Lymphocytes (×10^9^/L)	0.97 ± 0.20	1.48 ± 0.32	<0.01
Platelets (×10^9^/L)	227.1 ± 34.64	206.81 ± 26.39	<0.01
SII (×10^9^/L)	2,072.56 ± 653.31	783.82 ± 315.38	<0.01

To further characterize the longitudinal changes in SII, a linear mixed-effects model was performed (Table 3). The analysis demonstrated significant main effects of group (*β* = −1,288.74, *t* = −15.53, *p* < 0.001) and time (Day 1 vs. Day 7: *β* = −157.55, *p* < 0.001; Day 3 vs. Day 7: *β* = 400.56, *p* < 0.001), indicating that SII differed significantly between groups and changed dynamically over time. Furthermore, a significant group-by-time interaction was observed on Day 3 (*β* = −230.58, *t* = −4.57, *p* < 0.001), suggesting that patients with high baseline SII exhibited a more pronounced inflammatory response during the early post-hemorrhagic period. These findings indicate that systemic inflammation after brainstem hemorrhage follows a distinct temporal pattern, characterized by an early increase, a peak approximately 72 h after onset, and a gradual decline thereafter.

**Table 3:** Linear mixed-effects model for longitudinal changes in SII.

DV: SII	*β*	*t*	*p*-value
Fixed effects			
Group (low vs. high SII)	−1,288.74	−15.53	<0.001
Time (Day 1 vs. Day 7)	−157.55	−4.41	<0.001
Time (Day 3 vs. Day 7)	400.56	11.22	<0.001
Group × time (Day 1)	52.24	1.04	0.302
Group × time (Day 3)	−230.58	−4.57	<0.001
Random effects (subject ID)			
Variance	120,457.29		
Standard deviation	347.07		
Repeated covariance parameters			
Residual variance	44,610.27		
Covariance	75,847.02		

### 3.3 Hematoma volume

**Figure 3:**
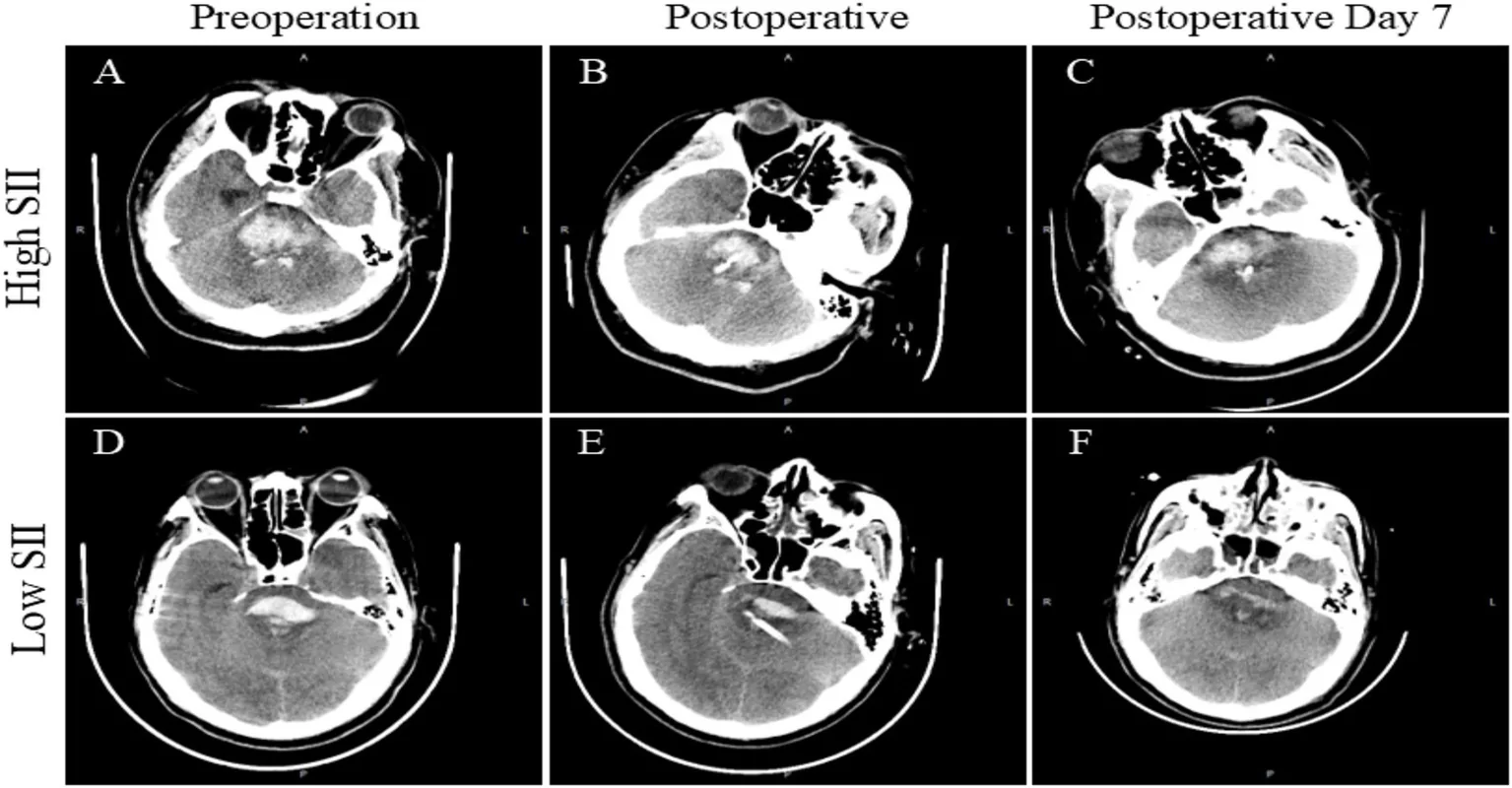
Representative axial brain CT images from patients stratified by high or low systemic SII levels. **(A–C)** Images from a patient in the high SII group, acquired preoperatively, postoperatively, and on postoperative day 7. **(D–F)** Corresponding time-point images from a patient in the low SII group.

Significant differences in hematoma volume distribution were observed between the high and low SII groups. In the high SII group, 33 patients (47.1%) presented with a hematoma volume <5 mL, compared with 46 patients (65.7%) in the low SII group (*p* = 0.027). For patients with hematoma volumes between 5 and 10 mL, the proportions were 31 (44.3%) and 24 (34.3%) in the high and low SII groups, respectively, with no statistically significant difference (*p* = 0.226). Notably, hematoma volumes ≥10 mL were identified in 6 patients (8.6%) in the high SII group, whereas no such cases occurred in the low SII group (*p* = 0.012; Table 4, Figure 3). Overall, patients in the high SII group tended to exhibit larger hematoma volumes, while smaller hematomas were more common in the low SII group.

**Table 4:** Hematoma volume.

Hematoma volume	High SII	Low SII	*p*-value
HV ≤ 5 mL	33 (47.1%)	46 (65.7%)	0.027
5 < HV ≤ 10 mL	31 (44.3%)	24 (34.3%)	0.226
HV > 10 mL	6 (8.6%)	/	0.012

**Figure 4:**
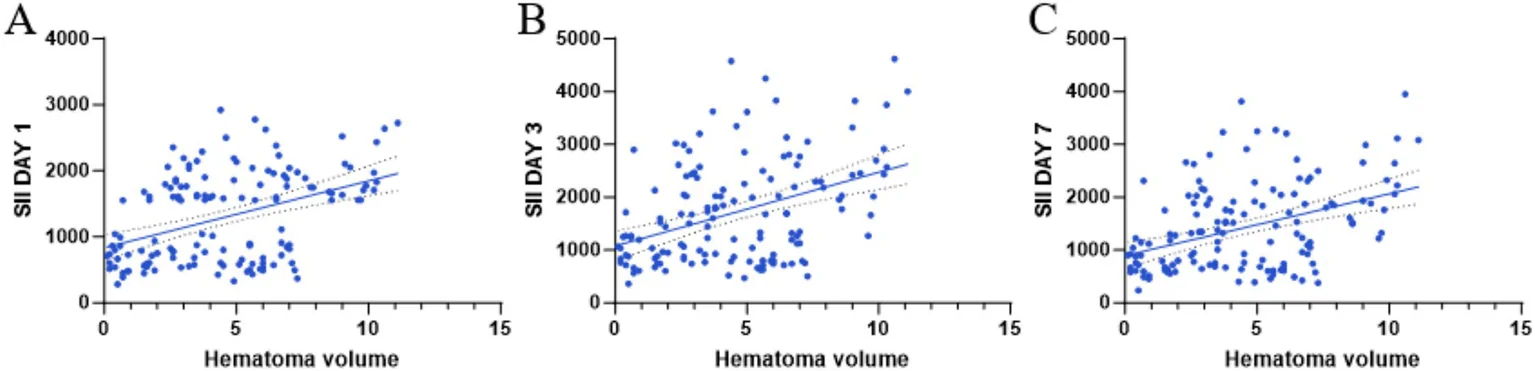
Spearman correlation analysis between hematoma volume and SII at Days 1, 3, and 7. **(A)** SII Day 1. **(B)** SII Day 3. **(C)** SII Day 7.

To investigate the relationship between hemorrhage severity and systemic inflammatory response, Spearman correlation analysis was performed between hematoma volume and SII at all measured time points: Day 1 (*ρ* = 0.379, *p* < 0.001), Day 3 (*ρ* = 0.356, *p* < 0.001), and Day 7 (*ρ* = 0.358, *p* < 0.001; Table 5, Figure 4). These findings indicate that larger hematoma volumes were associated with higher systemic inflammatory activity.

**Table 5:** Correlation analysis between hematoma volume and inflammatory biomarkers.

Variable	Spearman *r*	*p-*value
Day1 SII	0.378	<0.001
Day3 SII	0.356	<0.001
Day7 SII	0.358	<0.001

### 3.4 Clinical outcomes and complications

The study demonstrated that patients in the high-SII group experienced significantly worse outcomes and a greater disease burden. As shown in Table 3, at the 90-day follow-up, the high-SII group exhibited markedly higher mRS scores compared with the low-SII group (4.61 ± 1.29 vs. 2.20 ± 0.93, *p* < 0.001). Additionally, the high-SII group had a significantly longer hospital stay (16.26 ± 3.51 days vs. 14.3 ± 4.56 days, *p* < 0.001) and a higher 30-day mortality rate (27.1% vs. 8.6%, *p* = 0.004). Regarding complications, the incidences of central fever (25.7% vs. 14.3%), pulmonary infection (28.6% vs. 21.4%), and hyponatremia (15.7% vs. 11.4%) were all significantly higher in the high-SII group compared with the low-SII group (all *p* < 0.001; Table 6). Taken together, these findings indicate that patients with brainstem hemorrhage who present with elevated SII early in the disease course exhibit poorer long-term neurological recovery, prolonged hospitalization, higher mortality risk, and an increased incidence of common complications, suggesting that an early pronounced systemic inflammatory response is closely associated with more severe clinical outcomes.

**Table 6:** Clinical outcomes and complications.

Outcome	High SII	Low SII	*p*-value
Length of hospital stay	16.26 ± 3.51	14.3 ± 4.56	<0.001
30-day mortality	19 (27.1%)	6 (8.6%)	0.004
mRS	4.61 ± 1.29	2.20 ± 0.93	<0.001
Adverse event			
Central fever	16 (25.7%)	12 (14.3%)	<0.001
Pulmonary infection	20 (28.6%)	17 (21.4%)	<0.001
Hyponatremia	11 (15.7%)	8 (11.4%)	<0.001

### 3.5 Multivariable logistic regression analysis

To determine whether SII is an independent predictor of clinical outcomes, multivariable logistic regression analyzes were performed adjusting for age, sex, admission GCS score, hematoma volume, hydrocephalus, systolic blood pressure, and hemorrhage location (pons as reference).

For 90-day poor functional outcome (mRS ≥ 3), both Day-1 and Day-3 SII remained independently associated with poor prognosis after adjustment for potential confounding factors (Table 7). Day-1 SII was independently associated with an increased risk of poor functional outcome (adjusted OR = 1.002, 95% CI: 0.999–1.004, *p* = 0.020), while Day-3 SII also remained a significant predictor (adjusted OR = 1.001, 95% CI: 0.999–1.002, *p* = 0.011). Other clinical variables, including age, admission GCS, hematoma volume, hydrocephalus, systolic blood pressure, sex, and hemorrhage location, were not independently associated with 90-day functional outcome.

**Table 7:** Multivariable logistic regression for 90-day poor outcome (mRS ≥ 3).

Variable	*β*	SE	Wald	Adjusted OR	95% CI	*p*-value
Age	−0.008	0.023	0.119	0.992	0.948–1.038	0.731
Male	−0.814	0.458	3.166	0.443	0.181–1.086	0.075
Admission GCS	0.127	0.089	2.028	1.135	0.953–1.352	0.154
Hematoma volume	−0.132	0.09	2.142	0.876	0.734–1.046	0.143
Hydrocephalus	0.874	0.547	2.555	2.397	0.821–7.002	0.110
Systolic BP	0.011	0.012	0.731	1.011	0.987–1.035	0.392
Day-1 SII	0.002	0.001	1.639	1.002	0.999–1.004	0.02
Day-3 SII	0.001	0.001	0.433	1.001	0.999–1.002	0.011
Pons-1	0.26	0.586	0.198	1.297	0.412–4.087	0.657
Midbrain-2	0.565	0.722	0.613	1.76	0.427–7.248	0.434

For 30-day mortality, Day-1 SII remained independently associated with mortality after multivariable adjustment (adjusted OR = 0.997, 95% CI: 0.994–1.000, *p* = 0.041). Moreover, Day-3 SII demonstrated an even stronger association with mortality (adjusted OR = 1.004, 95% CI: 1.002–1.006, *p* < 0.001). Among the conventional clinical variables, only admission GCS remained an independent predictor of 30-day mortality (adjusted OR = 1.307, 95% CI: 1.006–1.698, *p* = 0.045; Table 8).

**Table 8:** Multivariable logistic regression for 30-day mortality.

Variable	*β*	SE	Wald	Adjusted OR	95% CI	*p*-value
Age	−0.058	0.036	2.545	0.944	0.879–1.013	0.111
Male	0.196	0.624	0.099	1.217	0.358–4.137	0.753
Admission GCS	0.268	0.133	4.023	1.307	1.006–1.698	0.045
Hematoma volume	−0.05	0.125	0.161	0.951	0.745–1.215	0.689
Hydrocephalus	−0.174	0.746	0.054	0.84	0.195–3.627	0.816
Systolic BP	0.001	0.016	0.001	1.001	0.970–1.033	0.971
Day-1 SII	−0.003	0.002	3.268	0.997	0.994–1.000	0.041
Day-3 SII	0.004	0.001	12.684	1.004	1.002–1.006	<0.001
Pons-1	0.926	1.066	0.755	2.524	0.312–20.393	0.385
Midbrain-2	0.146	1.195	0.015	1.157	0.111–12.036	0.903

To evaluate whether sex modifies the prognostic value of SII, a sex × Day 3 SII interaction term was included in the multivariable model. The interaction term was not statistically significant for 30-day mortality (*p* for interaction = 0.682), indicating that the predictive value of SII is consistent across sexes. The descriptive statistics stratified by sex are presented in Supplementary Table S1.

### 3.6 ROC curve analysis

**Figure 5:**
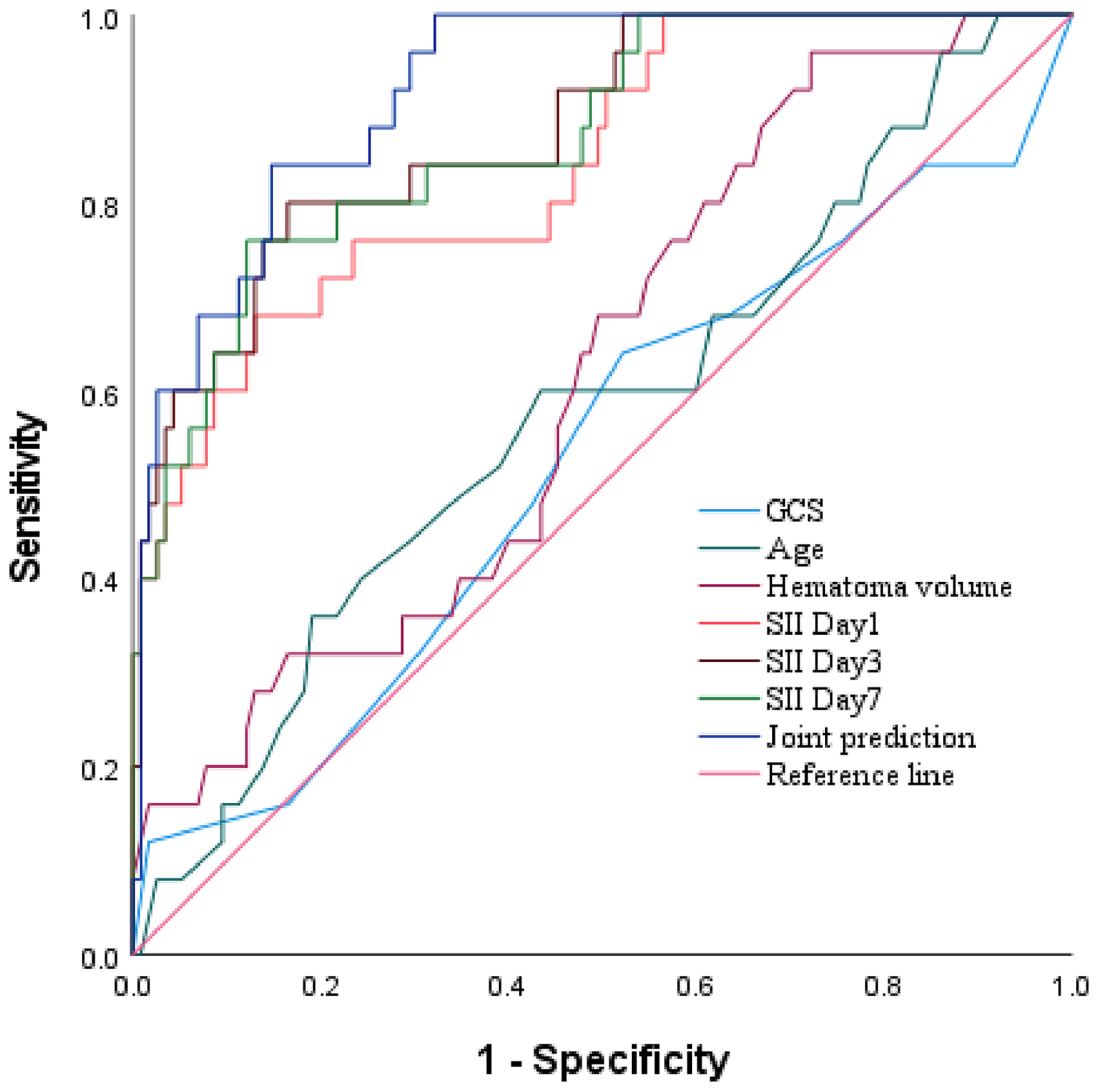
Predictive performance of SII for 30-day mortality in patients with brainstem hemorrhage.

ROC curve analysis demonstrated that SII measured on Day 1 after admission predicted 30-day mortality with an area under the curve (AUC) of 0.836 (95% CI, 0.752–0.920). The predictive performance of SII peaked on Day 3, with an AUC of 0.878 (95% CI, 0.804–0.952), and remained high on Day 7, with an AUC of 0.870 (95% CI, 0.793–0.947). The Youden index for Day 3 SII corresponded to an optimal cutoff value of 2,272 × 10^9^/L, which may serve as a reference for clinical risk stratification. Notably, the combined prediction model incorporating Day-1, Day-3, and Day-7 SII, GCS, age, and hematoma volume achieved the highest discriminatory performance (AUC = 0.920, 95% CI: 0.871–0.969), demonstrating that integrating inflammatory biomarkers with conventional clinical assessments substantially improves prognostic accuracy (Table 9, Figure 5).

**Table 9:** ROC analysis for predicting 30-day mortality.

Predictor	AUC	95% CI	Sensitivity	Specificity	Cutoff	*p*-value
GCS	0.526	0.394–0.657	0.000	1.000	4.000	0.689
Age	0.577	0.451–0.704	0.360	0.909	56.5	0.227
Hematoma volume	0.617	0.503–0.732	0.960	0.278	2.350	0.066
Day-1 SII	0.836	0.752–0.920	0.680	0.852	1,862.45	<0.001
Day-3 SII	0.878	0.804–0.952	0.800	0.835	2,271.73	<0.001
Day-7 SII	0.870	0.793–0.947	0.760	0.838	1,997.38	<0.001
Combined model	0.920	0.871–0.969	0.840	0.852	–	<0.001

## 4 Discussion

Our study systematically evaluated the dynamic changes in the SII during the acute phase of brainstem hemorrhage and explored their association with clinical outcomes. Our findings indicate that patients with elevated SII levels exhibited persistently heightened systemic inflammatory responses throughout the early hospitalization period, with a peak occurring on Day 3 after admission. Importantly, longitudinal analysis using a linear mixed-effects model confirmed significant differences in SII trajectories between high- and low-SII groups, suggesting that early systemic inflammatory activation follows a distinct temporal pattern after brainstem hemorrhage. High SII levels were significantly associated with poorer neurological recovery, higher mortality, longer hospital stays, and increased incidence of in-hospital complications, underscoring the prognostic significance of SII as an integrated biomarker reflecting systemic inflammation, immune suppression, and platelet activation.

From a pathophysiological viewpoint, secondary injury following brainstem hemorrhage is closely associated with the swift activation of inflammatory pathways and a disruption of immune equilibrium (27). Accumulating evidence indicates that neuroinflammation represents a central component of secondary brain injury after intracerebral hemorrhage, involving activation of microglia, infiltration of peripheral immune cells, cytokine release, and disruption of the neurovascular unit (28). Previous studies have reported that neutrophils are rapidly activated after acute brain injury, releasing reactive oxygen species, proteolytic enzymes, and neutrophil extracellular traps NETs, which contribute to blood–brain barrier disruption, cellular apoptosis, and microcirculatory impairment (29). In contrast, lymphopenia reflects systemic immune suppression and is associated with an increased risk of infection, fever, and metabolic dysregulation (30). Elevated platelet counts further promote coagulation activation and microthrombus formation, exacerbating ischemic injury in the brainstem (31). In our study, patients in the high-SII group consistently exhibited a pattern of elevated neutrophils, decreased lymphocytes, and increased platelet activation at all three time points. This characteristic change suggests that SII can effectively integrate multiple pathophysiological processes triggered by acute brainstem hemorrhage, including systemic inflammatory response, cellular immune suppression, and prothrombotic state, thereby serving as a comprehensive indicator that more fully reflects the severity of early secondary injury.

In addition to its connection with clinical outcomes, our study further showed that SII was positively correlated with hematoma volume on Days 1, 3, and 7 after admission. This ongoing correlation indicates that systemic inflammatory activation is closely tied to the severity of hemorrhage during the acute phase. Larger hematomas may cause greater mechanical compression, disrupt the blood–brain barrier, and activate resident microglia and peripheral immune cells, thereby intensifying systemic inflammatory responses. Previous research has indicated that hematoma volume is closely linked to inflammatory activation, perihematomal edema formation, and adverse neurological outcomes, suggesting that the burden of hematoma may act as a significant trigger for systemic immune responses following intracerebral hemorrhage (32). Conversely, excessive inflammatory activation may further aggravate secondary tissue injury through endothelial dysfunction, oxidative stress, and microvascular impairment. Our linear mixed-effects model further revealed that the variance of patient-specific random intercepts accounted for a substantial proportion of the total variability, emphasizing the marked inter-individual heterogeneity in inflammatory responses after brainstem hemorrhage. This finding supports the use of mixed-effects approaches for repeated-measures data and suggests that individualized inflammatory trajectories may provide additional prognostic information beyond single time-point measurements.

The bidirectional interaction between hematoma burden and systemic inflammation may therefore contribute to neurological deterioration after brainstem hemorrhage. A significant positive correlation was observed between hematoma volume and SII at all measured time points, raising the possibility that larger hematoma volume may drive a more robust systemic inflammatory response. Although SII remained independently associated with outcomes after adjustment for hematoma volume, the cross-sectional design precludes definitive conclusions regarding causality. The temporal pattern—with SII peaking on Day 3—suggests sustained inflammatory activation beyond the initial mechanical insult, yet reverse causality cannot be entirely excluded.

Notably, SII measured on Day 3 demonstrated the strongest predictive performance for 30-day mortality, with an AUC of 0.83 and an optimal cutoff value of 2,272 × 10^9^/L based on the Youden index. Furthermore, the combined prediction model achieved an even higher discriminative ability (AUC = 0.920), suggesting that integrating SII with conventional clinical variables may improve early mortality prediction. Patients exceeding this threshold had a significantly increased risk of poor prognosis. Research has shown that the inflammatory response following a stroke generally peaks within 48–72 h, and biomarkers assessed during this period offer the greatest prognostic value (33). In line with these findings, recent studies employing serial inflammatory measurements suggest that tracking dynamic changes, rather than relying solely on single baseline values, may more effectively capture the evolution of immune responses after acute brain injury and enhance outcome predictions (34, 35). Our findings further support this critical inflammatory window and demonstrate that serial SII monitoring provides additional information beyond a single baseline measurement. The superior performance of Day-3 SII may reflect the transition from the initial systemic stress response to a sustained inflammatory amplification phase, during which immune dysregulation and secondary injury mechanisms become more prominent.

The independent prognostic value of SII was further validated through multivariable logistic regression analyzes. After adjusting for established prognostic factors—including admission GCS, hematoma volume, hydrocephalus, age, sex, systolic blood pressure, and hemorrhage location—Day-3 SII remained an independent predictor of both 90-day poor functional outcome and 30-day mortality. Notably, admission GCS was independently associated only with 30-day mortality but not with 90-day functional outcome in our cohort, suggesting that SII may reflect dynamic pathophysiological changes that extend beyond baseline clinical severity. Analysis by sex showed no significant interaction between sex and SII, indicating that the prognostic value of SII is consistent across male and female patients. This finding reinforces the robustness of our main conclusions and supports the use of SII as a sex-neutral biomarker in clinical practice.

High SII levels were significantly linked to poorer neurological outcomes, as well as increased rates of pulmonary infections, central fever, and hyponatremia. These complications may stem from immune suppression reflected by SII, where lymphocyte depletion diminishes immune surveillance and makes patients more susceptible to infections (36). Additionally, inflammatory cytokines and hypothalamic dysregulation could play a role in central fever, while inflammatory cascades might impact the hypothalamic–pituitary–adrenal axis and antidiuretic hormone secretion, leading to electrolyte disturbances (37). We recognize that infections occurring before Day-3 blood sampling may elevate SII levels, potentially introducing reverse causation. Therefore, Day-3 SII should be viewed as indicative of the overall inflammatory burden rather than solely reflecting hemorrhage-induced inflammation. Future prospective studies with ongoing infection monitoring are necessary to clarify the temporal relationship between infection and SII elevation. Previous research on stroke and acute brain injury has also highlighted similar links between early inflammatory activation and complication risk, reinforcing the importance of SII as a prognostic indicator.

Our study provides several innovative contributions, including a comprehensive characterization of early SII dynamics in brainstem hemorrhage and an integrated analysis of its relationship with both prognostic outcomes and major complications. However, certain limitations should be acknowledged. This study was a single-center retrospective analysis, and potential selection bias cannot be entirely excluded. While the sample size is adequate, it may not fully capture the diversity of brainstem hemorrhage. Additionally, radiological markers of secondary injury, such as quantitative MRI parameters or inflammatory imaging biomarkers, were not included in the analysis. Moreover, although SII reflects systemic inflammatory status, direct measurements of inflammatory mediators, immune cell subsets, and molecular mechanisms were unavailable, preventing further mechanistic validation. Furthermore, treatment strategies may have influenced both inflammatory responses and clinical outcomes. In patients with brainstem hemorrhage, therapeutic decisions are usually individualized according to neurological status, hematoma characteristics, changes in pressure changes, and clinical progression. However, because detailed treatment-related information, including the exact distribution of surgical interventions and specific surgical procedures, was not systematically collected in our retrospective database, we were unable to fully evaluate whether treatment strategies differed between SII groups or completely adjust for their potential effects in the statistical models. Therefore, differences in therapeutic approaches may represent an unmeasured confounding factor influencing the association between SII and clinical outcomes. Future prospective studies with standardized treatment protocols, detailed recording of surgical interventions, and comprehensive adjustment for treatment-related variables are warranted to further validate the independent prognostic value of SII. In addition, although multivariable analyzes were performed to adjust for several established prognostic factors, including age, neurological severity, hematoma volume, and hydrocephalus, residual confounding factors may still exist due to the observational study design. Therefore, the prognostic role of SII should be interpreted as an association rather than a definitive causal predictor of adverse outcomes. Future multicenter prospective studies that incorporate multimodal imaging and mechanistic validation are needed to further investigate the clinical utility of SII and determine whether anti-inflammatory or immunomodulatory treatments could enhance outcomes for patients with high SII.

Furthermore, treatment strategies may have influenced both inflammatory responses and clinical outcomes. In patients with brainstem hemorrhage, treatment decisions are typically tailored to individual neurological status, characteristics of the hematoma, changes in intracranial pressure, and clinical progression. However, our retrospective database did not systematically collect detailed treatment-related information, including the specific distribution of surgical interventions and procedures. As a result, we could not fully assess whether treatment strategies varied between SII groups or adequately adjust for their potential effects in our statistical models. Consequently, variations in therapeutic approaches may serve as an unmeasured confounding factor affecting the relationship between SII and clinical outcomes. Future prospective studies with standardized treatment protocols, thorough documentation of surgical interventions, and comprehensive adjustments for treatment-related variables are necessary to further confirm the independent prognostic value of SII.

In conclusion, SII serves as a valuable biomarker that reflects systemic inflammatory activation and immune dysregulation in patients with brainstem hemorrhage. Early SII elevation—especially the peak observed on Day 3—is strongly associated with 90-day neurological outcomes, mortality, hospitalization duration, and complication risk. These findings support the use of SII for early risk stratification and individualized management strategies in brainstem hemorrhage. Future studies should validate these findings and investigate whether incorporating inflammatory modulation into clinical management can improve outcomes in this vulnerable patient population.

## Data Availability

The raw data supporting the conclusions of this article will be made available by the authors, without undue reservation.
