# Supplementary material for: Early systemic immune-inflammation index is associated with mortality and functional outcome in brainstem hemorrhage
Source: Front Neurol. 2026 Aug 26;17:1879981. doi: 10.3389/fneur.2026.1879981 (PMC13553423; doi:10.3389/fneur.2026.1879981)
Supplement: Supplementary file 1 [file Supplementary_file_1.docx]

Early Systemic Immune-Inflammation Index Is Associated with Mortality and Functional Outcome in Brainstem Hemorrhage

Song He1,2, Youkun Zhang1,2, Wenfeng Wei3, Huiyang Shi1,2, Rong Qin1,2, Zhenxing Yu1,2, Xianbing Zhang1,2, Lei Deng1,2*, Yongjie Zou1,2*

1. Department of Neurosurgery, No 908th Hospital of Chinese PLA Joint Logistic Support Force, Nanchang, Jiangxi 330002, China

2. Department of Neurosurgery, Changcheng Hospital Affiliated to Nanchang University, Nanchang, Jiangxi 330002, China

3. Department of Radiology, No 908th Hospital of Chinese PLA Joint Logistic Support Force, Nanchang, Jiangxi 330002, China

*Corresponding author: Yongjie Zou, E-mail: yongjiezou@163.com

Lei Deng, E-mail: denglei908@163.com

Supplementary Table S1. Gender-stratified descriptive statistics of major variables

| Variable | Male (n = 66) | Female (n = 74) | t | p |
| --- | --- | --- | --- | --- |
| SII Day1 | 0.45± 0.50 | 0.54 ± 0.50 | −1.012 | 0.313 |
| mRS≥3 | 0.62 ± 0.49 | 0.76± 0.43 | -1.742 | 0.084 |
| 30-day mortality | 0.19 ± 0.39 | 0.17 ± 0.38 | 0.094 | 0.925 |
